# Modulation of COUP-TF Expression in a Cnidarian by Ectopic Wnt Signalling and Allorecognition

**DOI:** 10.1371/journal.pone.0019443

**Published:** 2011-04-28

**Authors:** David J. Duffy, Uri Frank

**Affiliations:** School of Natural Sciences and Martin Ryan Marine Science Institute, National University of Ireland Galway, Galway, Ireland; National Institute on Aging Intramural Research Program, United States of America

## Abstract

**Background:**

COUP transcription factors are required for the regulation of gene expression underlying development, differentiation, and homeostasis. They have an evolutionarily conserved function, being a known marker for neurogenesis from cnidarians to vertebrates. A homologue of this gene was shown previously to be a neuronal and nematocyte differentiation marker in *Hydra*. However, COUP-TFs had not previously been studied in a colonial cnidarian.

**Methodology/Principal Findings:**

We cloned a *COUP-TF* homologue from the colonial marine cnidarian *Hydractinia echinata*. Expression of the gene was analysed during normal development, allorecognition events and ectopic Wnt activation, using *in situ* hybridisation and quantitative PCR. During normal *Hydractinia* development, the gene was first expressed in post-gastrula stages. It was undetectable in larvae, and its mRNA was present again in putative differentiating neurons and nematocytes in post-metamorphic stages. Global activation of canonical Wnt signalling in adult animals resulted in the upregulation of *COUP-TF*. We also monitored a strong *COUP-TF* upregulation in stolons undergoing allogeneic interactions. *COUP-TF* mRNA was most concentrated in the tissues that contacted allogeneic, non-self tissues, and decreased in a gradient away from the contact area. Interestingly, the gene was transiently upregulated during initial contact of self stolons, but dissipated rapidly following self recognition, while in non-self contacts high expression levels were maintained.

**Conclusions/Significance:**

We conclude that *COUP-TF* is likely involved in neuronal/nematocyte differentiation in a variety of contexts. This has now been shown to include allorecognition, where *COUP-TF* is thought to have been co-opted to mediate allorejection by recruiting stinging cells that are the effectors of cytotoxic rejection of allogeneic tissue. Our findings that Wnt activation upregulates *COUP-TF* expression suggests that Wnts' role in neuronal differentiation could be mediated through *COUP-TF*.

## Introduction

**Figure 1:**
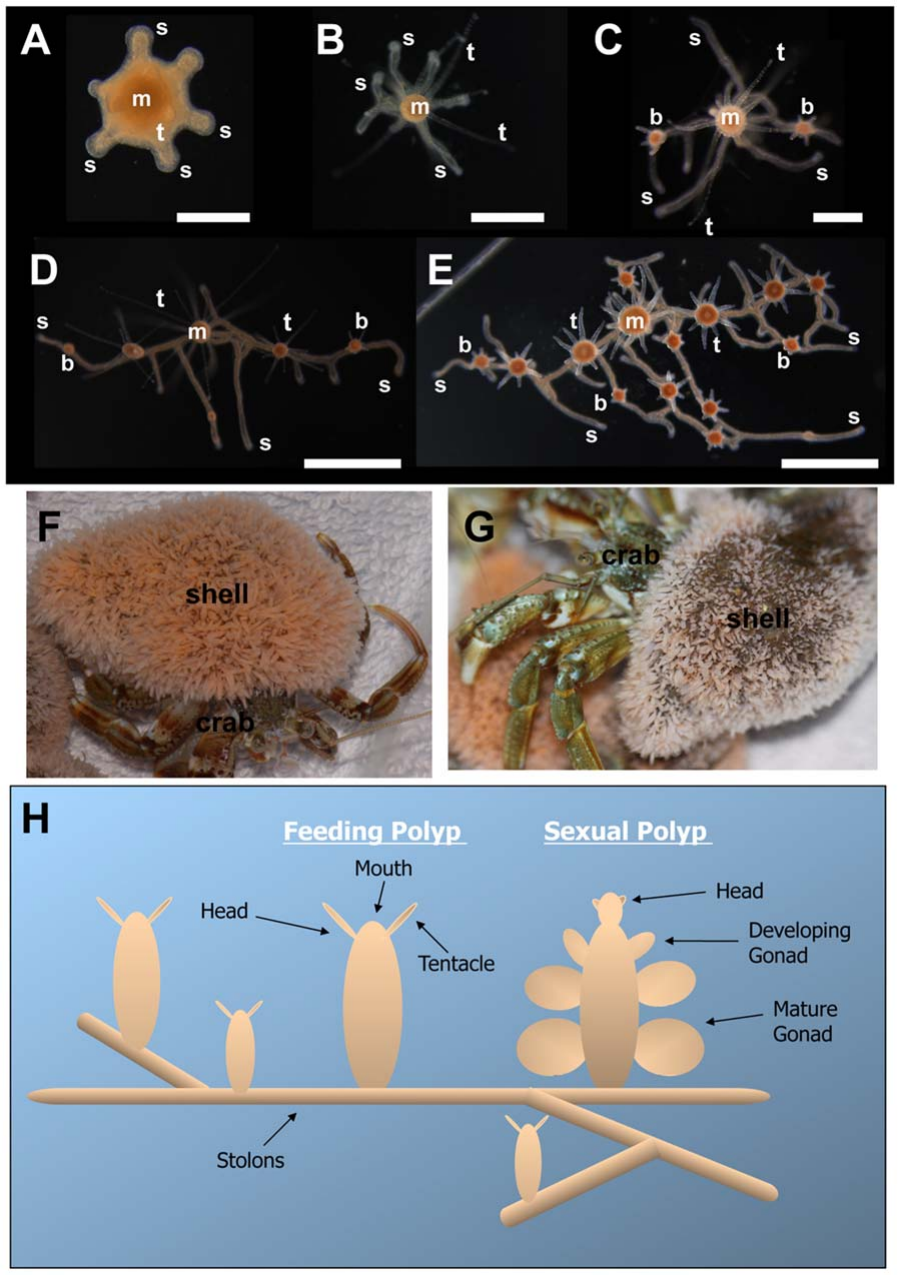
*Hydractinia echinata* colony structure. (**A–E**) Early colony growth, viewed from above. **m**; mouth of primary polyp. **t**; tentacle. **s**; stolon. **b**; newly budding polyp. (**A**) Developing primary polyp, one day after induction of metamorphosis, stolon and tentacles are starting to develop. (**B**) Polyp 2 days after induction of metamorphosis, stolons have grown longer. (**C**) Colony 5 days after induction of metamorphosis, new feeding polyps have budded from the stolons. (**D**) Colony 7 days after induction of metamorphosis, colony has continued to grow by stolon extension and budding of new polyps. (**E**) Colony 10 days after induction of metamorphosis consists of numerous polyps at various stages of development and an increasing stolon network. (**F, G**) Hermit crabs whose shells are encrusted by mature *Hydractinia* colonies, consisting of thousands of polyps and extensive stolon networks. (**H**) Schematic illustration of colony structure. Feeding polyps (gastrozooid) and sexual polyps (gonozooid) are linked by a system of gastrovascular canals called stolons. Stolons grow along the substrate budding new polyps at regular intervals. Scale bars (A) 200 µm, (B, C) 500 µm and (D, E) 1 mm.

*Hydractinia echinata* is a dioecious colonial marine organism belonging to the phylum Cnidaria. Colonies are most often found encrusting hermit crab shells (Figure 1). They are composed of repeating, genetically identical but functionally distinct polyps. Sexual reproduction occurs daily with light-induced spawning events during which eggs and sperm are released into the water by female and male colonies, respectively. The embryo develops within three days into a planula larva that is competent to metamorphose [1]. After receiving an external metamorphosing signal, the larva attaches to the substrate and develops into a primary polyp. Polyps are polarised with a mouth surrounded by tentacles at the oral pole (referred to as the head) and stolons at the aboral pole. Colonies grow by elongation of the stolons, from which new clonal polyps bud (Figure 1). The stolons form a system of gastrovascular tubes that enables distribution of food and exchange of stem cells among remote parts of the colony [2], [3].

Stolons of young colonies often grow into contact with those of conspecifics following co-settlement of allogeneic larvae on the same shell, which they actively locate [4]. *Hydractinia* allorecognition has been investigated for over fifty years [5], [6]. The genetic basis of the recognition process, however, has only recently been elucidated and found to be determined by two co-dominantly expressed genes, *alr1* and *alr2*, which are located in one chromosomal region, called the allorecognition complex (ARC). Sharing of at least one allele at both gene loci results in permanent fusion and chimera establishment, whereas sharing of alleles at only one locus results in transitory fusion, followed by rejection. Colonies that share no allele at either locus will reject each other without fusion and will aggressively compete [7]–[13]. Downstream genes acting in *Hydractinia* allorejection have not yet been identified.

Colonies recruit extensive numbers of nematocytes to stolon contact sites [7], [8]. Nematocytes are cnidarian-specific mechanoreceptor cells that contain nematocysts: toxin containing, harpoon-like structures enclosed in minicollagen capsules which are used for defence and prey capture [7], [8], [12], [14]. Of the five nematocyte types present in *Hydractinia*, only one, the microbasic mastigophore, is involved in allogeneic interactions. These cells migrate to the contact site and discharge their nematocysts, inflicting damage to the foreign tissue [7], [8], [12]. Allogeneic conflicts can last for weeks with repeated nematocyst discharges from both colonies, until one colony has defeated its opponent. During the rejection response, stolons can become hyperplastic, rising off the substrate, growing over the opposing colony and swelling with nematocytes [15]. We show here that the allorecognition responses result in local upregulation of the *Hydractinia chicken ovalbumin upstream promoter transcription factor* (*COUP-TF*) gene, which is, to the best of our knowledge, the first identified effector gene in cnidarian allorecognition.

COUP-TFs belong to the steroid/thyroid hormone receptor superfamily of nuclear receptors (NR) and are required for regulation of gene expression underlying development, differentiation, and homeostasis [16], [17]. COUP-TFs are also known as nuclear receptor subfamily 2, group F (NR2F) genes. COUP-TFs bind to evolutionarily conserved DNA motifs in their target genes either as homodimers, or as a heterodimer with retinoid X receptors (RXRs), and generally behave as a potent negative transcriptional regulator, although they can also act as transactivators [18]–[23]. COUP-TF (NR2F) is a known marker for neurogenesis from cnidarians to vertebrates, and *Hydra COUP-TF* has been shown to be expressed during both neuronal and nematocyte differentiation, where it is thought to promote entry into the differentiation process [24]–[27]. Cnidarian COUP-TFs have also been identified in corals and sea anemones [28], [29].

Nematocytes originate from the neuronal lineage. Nematogenesis has been primarily studied in *Hydra* and *Clytia*. In *Hydra* nematocytes differentiate throughout the body column ectoderm, then migrate to the tentacles [30], [31]. In contrast, nematogenesis in the medusa stage of *Clytia* is restricted to the tentacle bulb [32]. We have isolated a *Hydractinia COUP-TF* gene whose expression is induced by inter-colony interaction, resulting in nematogenesis.

Wnt signalling in *Hydractinia* has been shown to result in the expansion of stem cells, which subsequently differentiate to nerve cells and nematocytes [33]. In other metazoans, self-renewal and expansion of stem cells have also been linked with Wnt/ß-catenin activation, in particular the expansion of neural progenitors [34]–[36], the self-renewal of haematopoietic stem cells [37], [38], and the maintenance of pluripotency of embryonic stem cells [39]. Wnt signalling has also been shown to be involved in the specification of cell fate in guiding neural crest stem cells out of the cell cycle into terminal differentiation [40]–[43]. Our results show that *COUP-TF* is a Wnt target gene, which is upregulated in response to Wnt signalling.

We have successfully isolated and characterised a *Hydractinia COUP-TF* gene and examined its expression in response to ectopic Wnt signalling and allorecognition events. These results have relevance for understanding the effector mechanisms of the Cnidarian rejection response and more broadly the evolutionary origin of the interactions between Wnt signalling and COUP-TF in neurogenesis.

## Results

### Gene sequences

**Figure 2:**
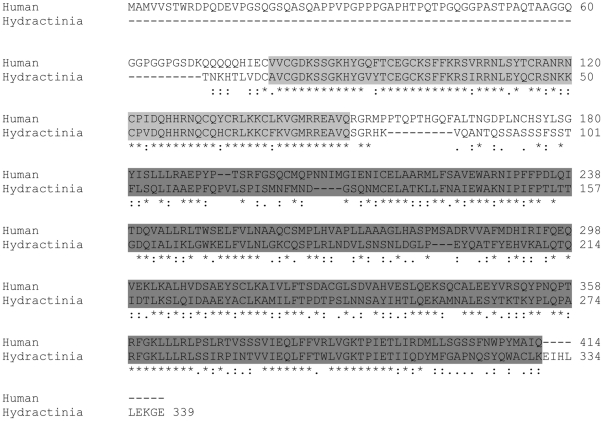
Alignment of the partial *Hydratinia echinata* COUP-TF coding sequence with its closest human match, COUP transcription factor 2 isoform a, accession number NP_066285. The light grey highlighted sequence corresponds to the conserved DNA-binding domain and the dark grey to the conserved ligand binding domain of COUP-TFs.

A fragment of 257 bp of the *COUP-TF* transcript was obtained from the SSH screen for genes upregulated by Wnt signalling. The remainder of the 3′ sequence to the polyA tail was then obtained by RACE PCR. In total, a partial sequence consisting of 1,239 bp of the *COUP-TF* mRNA transcript was obtained, which represented a 339 amino acid coding sequence – corresponding to almost the full human protein. The sequence was deposited in GenBank under accession number JF414805. BLAST analysis of the predicted amino acid sequence revealed that the four closest sequences were *Cupiennius salei* seven-up (NR2F3), a predicted protein from *Hydra magnipapillata*, the *Mus musculus COUP-TFI*, and *Homo sapiens* COUP transcription factor 2 isoform a (NR2F2) (GenBank accession numbers CAH59197, XP_002159396, XP_001475509 and NP_066285, respectively). Another similar cnidarian sequence was *Acropora millepora* nuclear receptor AmNR7 (GenBank accession number AAL29200). The *Hydractinia* COUP-TF predicted amino acid sequence showed 51% identity to the overall human sequence with higher rates in the conserved DNA-binding domain and the ligand-binding domain (Figure 2).

### Expression pattern of *COUP-TF* during development

**Figure 3:**
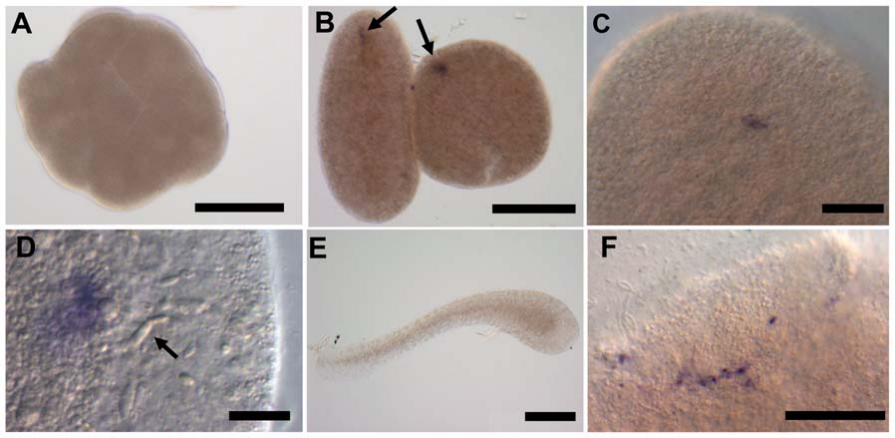
*COUP-TF* expression during development. (**A**) Early embryo: no expression is detectable by *in situ* hybridisation in the first 24 hours of embryonic development. (**B**) A spot of expression appears in pre-planula (approximately 24 hours post-fertilisation). (**C, D**) Higher magnification of pre-planula expression spots. (**C**) A single spot of expressing cells appears at one pole of the pre-planula only. (**D**) Nematocytes are visible to the right of the expression site (arrow). (**E**) No expression is detectable in planula larvae. (**F**) Putative *COUP-TF* expressing nerve cells are detectable in primary polyps. Scale bars (A, B & E) 200 µm, (C & F) 50 µm and (D) 20 µm.

Similar to *Hydra*, *COUP-TF* expression was not detectable throughout cleavage by *in situ* hybridisation [24]. As of the late post-gastrula stage (pre-planula), a spot of *COUP-TF* expressing cells became evident (Figure 3B–D). This is the proposed location of nerve cells and nematocyte precursors at this stage in *Podocoryne* larvae, a closely related hydrozoan [26]. However, *COUP-TF* expression was not detectable in planula larvae. *COUP-TF* expression did return after metamorphosis, in putative differentiating neuronal cells, near the head of primary polyps, which is the pole corresponding to the pre-planula expression spot (Figure 3). This is also the same pole which expresses *Wnt3* [44]–[46].

### *COUP-TF* and allorecognition

**Figure 4:**
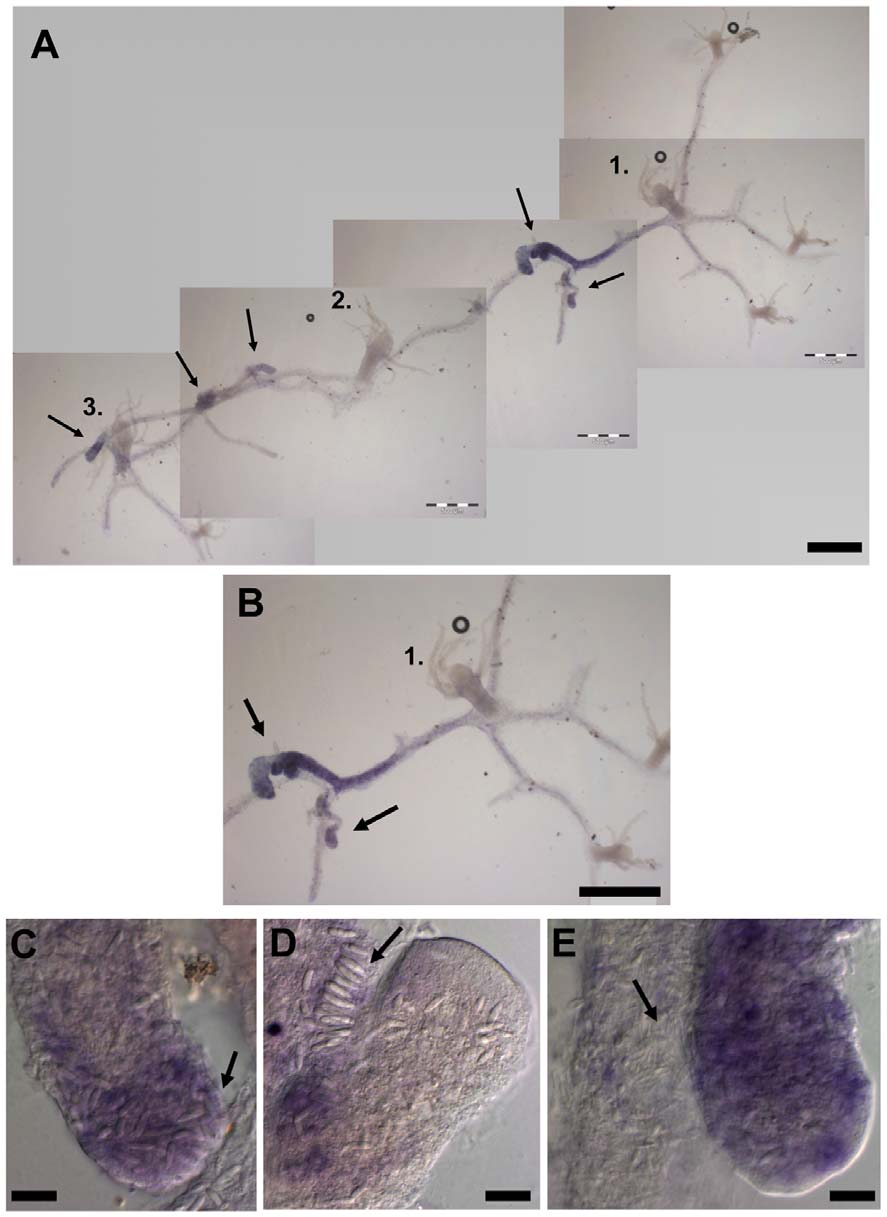
*COUP-TF* mRNA expression during allogeneic colony rejection. (**A**) Three young colonies whose stolons have grown into contact. Original primary polyps of each colony are numbered. Arrows indicate colony stolon contact sites and corresponding *COUP-TF* expression. (**B**) Close-up of contact sites between colony 1 and 2 (arrows) and *COUP-TF* expression. Expression is strongest in contact regions, and decreases away from the contact zone in a graded manner. The upper contacting stolon of colony 1 is clearly hyperplastic, being swollen and overgrowing its competitor. (**C–E**) Higher magnification views of competing stolons. Nematocyte build up (differentiation to discharge). (**C**) Increased expression corresponds to nematocyte differentiation. Mature nematocytes are visibly swelling the upper stolon tip (arrow). Nests of differentiating *COUP-TF* expressing nematoblasts are also visible. (**D**) A later stage contact site. The right stolon has overgrown the left one. Differentiated nematocytes of left stolon (arrow) are lining up in a battery in preparation for discharge. (**E**) Close-up of the lower contact site in image B. Arrow indicates large numbers of nematocytes. Scale bars (A & B) 500 µm, (C–E) 20 µm.

While performing *in situ* hybridisation with young colonies, we noticed high *COUP-TF* expression in the interface between two allogeneic colonies. To examine this phenomenon in more detail, colonies were allowed to grow into contact with one another before being fixed for *in situ* hybridisation. During allogeneic rejection, the level of *COUP-TF* expression increased significantly at stolon contact sites (Figure 4). It was also strongly upregulated throughout large parts of the adjacent stolonal network in a graded manner away from the contact site (Figure 4). It remains to be confirmed whether the COUP-TF protein is co-located with its mRNA. *COUP-TF* expressing stolons contained large numbers of differentiated nematocytes; such cells are clearly visible in the expressing tissue (Figure 4C–E). Later-stage hyperplastic stolons, identified by their growth over adjoining competitor colonies, showed very strong *COUP-TF* expression and were packed with nematocytes. Fully differentiated nematocytes showed no *COUP-TF* expression (Figure 4C–E) consistent with the case in *Hydra* [24].

### Self Recognition

**Figure 5:**
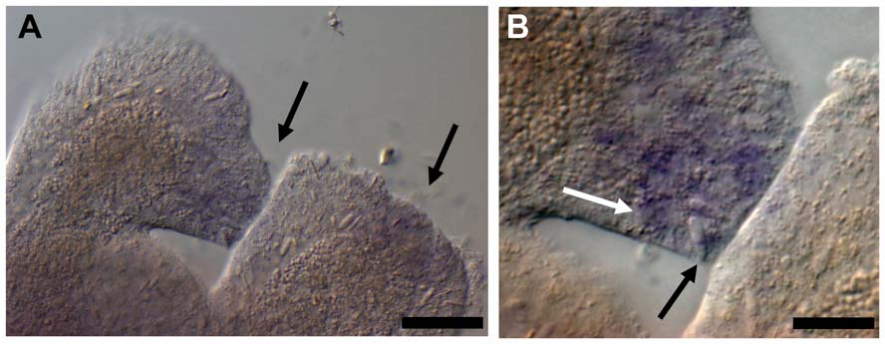
*COUP-TF* expression during stolon self contact in a single primary polyp, one day after metamorphosis. (**A**) The left stolon has grown into contact with the right stolon. Expression is visible at the contact site of the left stolon and at the tip of the right stolon, adjacent to the contact site (arrows). (**B**) Higher magnification view of contact site. White arrow indicates putative nematoblast, black arrow indicates nematocyte. Scale bars (A) 50 µm and (B) 20 µm.

*COUP-TF* was also transiently upregulated during early stages of self-stolon encounters (Figure 5), but was downregulated shortly after contact and not seen in later-stage fused stolons. This is consistent with previous research [8], which showed that even self encounters result in initial recruitment of microbasic mastigophores to the predicted contact area before self recognition and dispersal.

### *COUP-TF* is upregulated following Wnt activation

**Figure 6:**
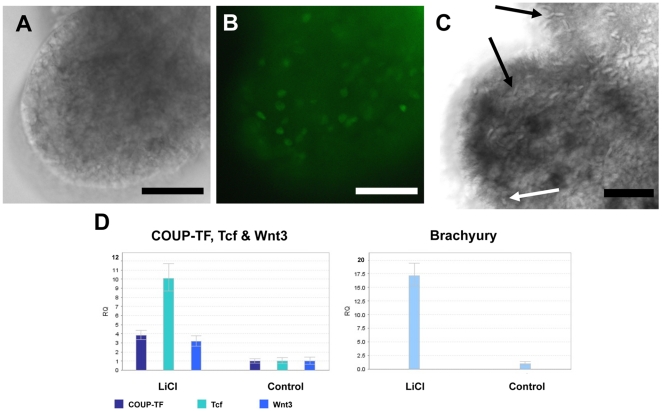
Proliferation, nematoblast differentiation and altered gene expression in response to Wnt activation. (**A, B**) Wnt induced outgrowth. (**A**) Bright field view of outgrowth. (**B**) Wnt induced outgrowths consist of numerous proliferating cells, as shown by BrdU labelling of proliferating nuclei (green fluorescence). (**C**) Nematocytes (black arrows) and nematoblast nests (white arrow) are visible in outgrowth of LiCl-treated polyp. (**D**) Expression levels of *COUP-TF* and known Wnt targets *Tcf*, *Wnt3* & *Brachyury* analysed by qPCR. Expression levels normalised to *GAPDH*. The relative quantity (RQ) of expression of each gene in control larvae were compared to expression levels in the 18.5 hour LiCl-treated pre-planula. Scale bars 50 µm.

We originally identified *COUP-TF* from a SSH screen of genes upregulated in response to ectopically activating Wnt signalling. In the screen, mRNA from LiCl-treated polyps and untreated controls were subtracted. LiCl is known to block GSK3 mediated β-catenin degradation, thereby mimicking the effect of Wnt ligand binding and activating Wnt target genes [33], [45]–[47]. The LiCl treatment resulted in ectopic outgrowths along the polyp body columns. These outgrowths were shown to contain proliferating cells (Figure 6A, B), consistent with the known role of Wnt signalling in *Hydractinia* [33]. They also contained nematoblast nests and differentiated nematocytes (Figure 6C). qPCR confirmed that *COUP-TF* expression increases in response to LiCl treatment, with LiCl-treated pre-planula showing a 3.8 fold increase of expression (Figure 6D). LiCl treatment also increased expression of *Brachyury* (17 fold), *Tcf* (10 fold) and *Wnt3* (3.2 fold), all of which are known Wnt target genes in *Hydractinia* [46].

## Discussion

We have shown here a hitherto undescribed link between Wnt signalling and *COUP-TF* expression in *Hydractinia*. Evidence from the literature suggests that this link is evolutionarily conserved across the metazoa, and may have broader implications: *COUP-TF* is upregulated in colorectal cancers [23], which also deregulate Wnt signalling [48], [49]. Upregulation of *COUP-TF* expression has been detected in other mammalian tumour tissue types. For example, it has been shown that ectopic expression of *COUP-TFII*, one of the two mammalian COUP-TFs, in human lung cancer cells results in the acquisition of invasive and migratory abilities as well as in vitro tumorigenicity [50]. In addition, *COUP-TFII* has been shown to be upregulated in tumorigenic mouse cells, where it is a repressor of MHC class I transcription, therefore, potentially aiding these cells in evading the immune response [51]. Our findings of Wnt's ability to upregulate *COUP-TF* expression mirrors recent results in the differentiation of human fat cells where canonical Wnt signalling activates the expression of *COUP-TFII*, resulting in the repression of adipogenesis [52]. These authors identified and functionally evaluated a Tcf binding site in the human *COUP-TFII* promoter and showed it to be a direct Wnt/β-catenin target in preadipocytes. Therefore, combined with our results, it appears that Wnt's ability to upregulate *COUP-TF* expression is ancient, having been established early in metazoan evolution.

The role of Wnt signalling in the control of vertebrate neurogenesis has recently been subject to further elucidation [41]–[43]. *Wnt3a* has been shown to promote hippocampal neurogenesis by shortening the cell cycle duration of mouse neural progenitor cells [43]. It remains to be discovered which Wnt ligand is responsible for increasing stem cell proliferation in cnidarians. It would also be of interest to further investigate whether the links shown previously between *Hydractinia* Wnt activation and subsequent neurogenesis and nematogenesis [33] (which our results indicate may be *COUP-TF* mediated) have similar underlying mechanisms to those emerging in vertebrate systems.

Our *COUP-TF* expression results during the rejection response address the query raised by Lange et al. [8] of whether increased proliferation and differentiation of precursor cells to microbasic mastigophores occurs locally in response to contact, or if differentiated microbasic mastigophores are attracted from distant stolon parts. The *COUP-TF* expression pattern indicates that the former option is more likely, with an increase in *COUP-TF* mediated differentiation close to the contact site, while the greater the distance from the site of contact, the weaker the *COUP-TF* expression. However, while this does not fully rule out immigration of differentiated nematocytes, the weaker distal expression of *COUP-TF* suggests that distant areas are not the primary suppliers of microbasic mastigophores to the contact zone. Distal areas may still be a source of stem cells, which undergo nematogenesis locally mediated by COUP-TF. These results add stolon contact sites, both self and non-self, to the known locations of nematogenesis in cnidarians, i.e. the *Hydra* body column and *Clytia* tentacle bulbs [30]–[32].

The expression pattern of *COUP-TF* suggests that its initial upregulation is unrelated to allorecognition. This is because even isogeneic (i.e. self) encounters result in initial *COUP-TF* induction and microbasic mastigophore recruitment to the contact zone [8]. The difference between compatible and incompatible contacts are that in the case of compatible touching stolons, *COUP-TF* expression dissipates and the nematocytes disperse, while incompatible interacting stolons continue to express high levels of *COUP-TF* and produce, or attract, large numbers of microbasic mastigophores, which are the effector mechanisms in the rejection process. The unusual stolon type produced in *Hydractinia* during allorejection, the hyperplastic stolon [15], also strongly expressed the gene. Therefore, we propose that initial *COUP-TF* expression in the contact zone is mediated by a non-allotypic diffusible factor emitted upon stolon contact (self or non-self), with *alr1/alr2* mediated recognition subsequently modulating *COUP-TF* expression, either downregulating it (in self recognition) or further upregulating it (in non-self recognition). It would be of interest to determine how *COUP-TF* transcriptional regulation is achieved, and whether it involves Wnt signalling, another regulator of *COUP-TF* expression reported here. Determining which specific portions of the *COUP-TF* promoter sequence are responsive to stolon contact and ARC-mediated allorecognition would likely prove informative in deciphering the intermediary signalling pathways which affect allogeneic rejection in cnidarians.

In summary, *Hydractinia* is a valuable model for examining the evolutionary origins of *COUP-TF* function in neurogenesis. Since neurogenesis/nematogenesis has been co-opted to the cnidarian allorejection mechanism, *COUP-TF* is unlikely to have a conserved role in allorecognition in other animals. The link between Wnt signalling and *COUP*-TF, reported here, may also provide insight into both stem cell and cancer biology.

## Materials and Methods

### Animals

*Hydractinia echinata* colonies were collected from Galway Bay by SCUBA divers. They were cultured in natural seawater at 18°C under 14/10 light-dark regimes and were fed brine shrimp nauplii (*Artemia salina*) six times a week. Sperm and eggs were collected daily about an hour after the onset of light. Embryos were kept in Petri dishes for three days until embryonic development was completed to metamorphosis-competent planula larvae. Metamorphosis was induced by a three-hour pulse treatment of 116 mM CsCl in seawater as previously described [53] and the animals were allowed to settle and metamorphose on glass coverslips. For LiCl experiments, polyps were cut from adult colonies near their bases using fine surgical scissors.

### Wnt target gene suppressive subtractive hybridisation (SSH)

Polyps were cut away from colonies at their base and incubated for 24 hours in seawater containing 28 mM LiCl. Control polyps were allowed to regenerate in normal seawater. Seventy five minutes after the treatment, mRNA from LiCl treated and control polyps was extracted. Total RNA was extracted by acid guanidinium/phenol:chloroform extraction. mRNA was purified from Total RNA using a Dynabeads mRNA Purification Kit (Dynal Biotech) according to manufacturer's instructions. These two mRNA populations were then used in a suppressive subtractive hybridisation (SSH) using a PCR-Select cDNA Subtraction Kit (Clontech Laboratories, Inc.) according to the manufacturer's protocol. The PCR products were then cloned using pGEM-T vector system (Promega). The subtracted library was sequenced and the differential expression of selected clones was verified by qPCR.

### Obtaining sequence data

A 257 bp fragment of the *COUP-TF* gene was obtained from the SSH for genes upregulated by mimicking Wnt signalling with LiCl (see above).

To obtain a longer sequence, Clontech's SMART RACE protocol was used for 3′ RACE. A *COUP-TF* specific forward primer was designed from the EST cloned sequence obtained from the SSH. Forward primer sequence: 5′GTCGATCAACATCACCGAAA3′. *ß-catenin* and *Tcf* sequences had been obtained previously [46] (GenBank accession numbers GU145277 and GU145278 respectively). *Brachyury*, *Wnt3*, and *GAPDH* primers were designed from the GenBank entries, accession numbers AF312733.1, AM279678 and DT622622, respectively.

### Quantitative, real-time PCR (qPCR)

Thirty-one-hour old embryos (pre-planula) were incubated in seawater containing 28 mM LiCl for 18.5 hours. After treatment, total RNA was extracted from samples to be analysed by acid guanidinium/phenol:chloroform extraction. The RNA was then DNase digested with RQ1 RNase free DNase (Promega) and phenol:chloroform purified. It was reverse transcribed to cDNA using Omniscript Reverse Transcriptase (Qiagen). qPCR was performed on a StepOne Plus (Applied Biosystems) with Power SYBR reagents according to the manufacturer's recommended protocol. Gene expression was normalised to the expression of *GAPDH*. Generated PCR products were analysed by melt curve analysis, by gel electrophoresis and by sequencing. The following primer sets were used: *GAPDH* nested forward 5′TGCTACAACTGCCACACAGAAAA3′ and reverse 5′CACCACGACCATCTCTCCATTT3′; *COUP-TF* forward 5′TGCGCAGTTTGTGGAGATAAA3′ and reverse 5′TGCAACCTTCGCATGTGTATACT3′; *Wnt3* forward 5′CCAACACCAACGCGAAGTATG3′ and reverse 5′ACCTTCCCCGACACTTCTGA3′; *Tcf* forward 5′GCGCCATTCACATGCAGTTA3′ and reverse 5′CGACCGATTTGTGCATAGTTGT3′; *Brachyury* forward 5′CCAACCGGCACCACTTAAA3′ and reverse 5′CGAGCTAACGGCGACACTTT3′.

### Allorecognition experiments

In order to examine gene expression during the allorecognition response, larvae were induced to metamorphose and were settled in close proximity to each other on a cover slip. These colonies were allowed to develop for a few weeks until their stolons grew into contact with one another. The colonies were then fixed and processed for *in situ* hybridisation while on the slide. To examine self contact, primary polyps whose own stolons had grown into contact with one another were similarly processed for *in situ* hybridisation.

### *In situ* hybridisation

*In situ* hybridisation was performed as previously described [54], [55]. Hybridisations were performed at 50°C. *COUP-TF* RNA probes were synthesised from a pGEM-T plasmid containing the 257 bp fragment of the gene obtained from the SSH.

### Bromodeoxyuridine (BrdU) proliferation assay

LiCl treated polyps (see above) were incubated in seawater containing 200 µM BrdU for 2 hours and thereafter washed with seawater. Forty minutes after the end of BrdU incubation they were fixed with paraformaldehyde. BrdU detection was performed as follows: The specimens were washed in PBS, then with 0.4 M Glycine pH7.2. Next they were treated with 2 M HCl, then washed with 0.25% Triton PBS. Unspecific binding of the antibodies was blocked with 1%BSA in 0.25% Triton PBS. Samples were then incubated for 1 hour at room temperature in 1∶500 dilution of anti-BrdU (Roche), then washed with 0.25% Triton PBS and incubated in the dark at room temperature for 1 hour in pre-absorbed 1∶500 dilution of secondary antibody, Alexa-Fluor 488 anti-mouse antibody (Invitrogen A11059). Finally they were washed with 0.25% Triton PBS.
